# Study protocol: NeoCLEAR: Neonatal Champagne Lumbar punctures Every time – An RCT: a multicentre, randomised controlled 2 × 2 factorial trial to investigate techniques to increase lumbar puncture success

**DOI:** 10.1186/s12887-020-02050-8

**Published:** 2020-04-15

**Authors:** Andrew S. J. Marshall, Manish Sadarangani, Alexandra Scrivens, Rachel Williams, Jean Yong, Ursula Bowler, Louise Linsell, Virginia Chiocchia, Jennifer L. Bell, Caz Stokes, Patricia Santhanadass, Eleri Adams, Edmund Juszczak, Charles C. Roehr, on behalf of the ‘The NeoCLEAR Collaborative Group’

**Affiliations:** 1 Department of Paediatrics, Oxford University Hospitals NHS Foundation Trust, John Radcliffe Hospital, Headley Way, Headington, Oxford, OX3 9DU UK; 2 Vaccine Evaluation Center, BC Children’s Hospital Research Institute, Vancouver, BC V5Z 4H4 Canada; 3 Department of Pediatrics, University of British Columbia, 4480 Oak St, Vancouver, BC V6H 0B3 Canada; 4 Newborn Care Unit, Oxford University Hospitals NHS Foundation Trust, John Radcliffe Hospital, Headley Way, Headington, Oxford, OX3 9DU UK; 5 National Perinatal Epidemiology Unit (NPEU) Clinical Trials Unit, Nuffield Department of Population Health, University of Oxford, Old Road Campus, Headington, Oxford, OX3 7LF UK; 6 Support for the Sick Newborn And their Parents (SSNAP) Charity, Level 2, The Women’s Centre, John Radcliffe Hospital, Oxford, OX3 9DU UK

**Keywords:** Neonate, Lumbar puncture, Meningitis, Preterm infant, Technique, Stylet

## Abstract

**Background:**

The neonatal period carries the highest risk of bacterial meningitis (~ 1 in 5000 births), bearing high mortality (~ 10%) and morbidity (20–50%) rates. Lumbar puncture (LP) remains essential to the diagnosis of meningitis. Though LP is a common procedure in neonates, success rates are lower (50–60%) than in other patient populations. None of the currently-practised neonatal LP techniques are supported by evidence from adequately-powered, randomised controlled trials (RCTs). NeoCLEAR aims to compare two modifications to the traditional technique which are free, accessible, and commonly practised: sitting (as opposed to lying) position, and ‘early’ (as opposed to ‘late’) stylet removal.

**Methods/design:**

Written parental informed consent permitting, infants in neonatal/maternity wards, of 27^+ 0^ to 44^+ 0^ weeks corrected gestational age and weighing ≥1000 g, who require an LP, will be randomly allocated to sitting or lying position, and to early or late stylet removal. The co-primary objectives are to compare success rates (the proportion of infants with cerebrospinal fluid red cell count < 10,000/mm^3^ on first LP procedure) in 1020 infants between the two positions, and between the two methods of stylet removal. Secondary outcomes relate to LP procedures, complications, diagnoses of meningitis, duration of antibiotics and hospital stay. A modified intention-to-treat analysis will be conducted.

**Discussion:**

Two modifications to the traditional LP technique (sitting vs lying position; and early vs late stylet removal) will be simultaneously investigated in an efficient and appropriately-powered 2 × 2 factorial RCT design. Analysis will identify the optimal techniques (in terms of obtaining easily-interpretable cerebrospinal fluid), as well as the impact on infants, parents and healthcare systems whilst providing robust safety data. Using a pragmatic RCT design, all practitioners will be trained in all LP techniques, but there will inevitably be variation between unit practice guidelines and other aspects of individual care.

An improved LP technique would result in:

• Fewer uninterpretable samples, repeated attempts and procedures

• Reduced distress for infants and families

• Decreased antibiotic use and risk of antibiotic resistance

• Reduced healthcare costs due to fewer procedures, reduced length of stay, shorter antibiotic courses, and minimised antibiotic-associated complications

**Trial registration:**

ISRCTN14040914. Date assigned: 26/06/2018.

## Background

Neonatal bacterial meningitis has high rates of mortality and morbidity in Europe and North America, and higher still in Africa and Asia [1]. The symptoms and signs are non-specific in neonates, and the diagnosis can only be confirmed by analysing cerebrospinal fluid (CSF), following lumbar puncture (LP). Infants with meningitis typically require 14–21 days of inpatient intravenous antibiotics, incurring significant financial costs, and often receive hospital follow-up due to the risk of long-term neurological sequelae [2]. Prolonged antibiotic use is associated with significant complications [3, 4]. If meningitis can be excluded, antibiotics are usually stopped after 5 days, allowing discharge with no further follow-up.

Each year up to 30,000 UK newborn infants undergo LP [5], but success rates are much lower in neonates (50–60%) [6, 7] than older children (78–87%) [8, 9]. Interpretation of non-clear CSF samples containing significant numbers of red blood cells (RBC) (> 5000, or > 10,000 RBC/mm^3^) is challenging and the presence/absence of meningitis difficult to confirm. Thus, LPs often need repeating, and lead to treatment with extended courses of antibiotics because meningitis cannot be excluded.

LP techniques currently vary by local practice. Several modifications to ‘traditional’ technique have been investigated, but few have good evidence of benefit. Two modifications identified during a literature review appeared the most promising and amenable to investigation in an RCT:

1. Sitting position, in which the infant is held in a semi-upright, sitting position compared to lying (‘lateral decubitus’) position.
2. ‘Early stylet removal’ (ESR), which is the removal of the stylet from the LP needle shaft once it has penetrated the subcutaneous tissue, and before advancing the needle into the CSF.

Sitting: the only published systematic review regarding LP in children/neonates examined sitting position and concluded that “Positions other than the lateral decubitus may be equal or superior in terms of lumbar puncture success” …and… “A large-scale prospective clinical trial directly addressing LP success and safety in different positions would clarify the need to change current practice” [10].

Stylet removal: traditional technique aims to insert the needle into the CSF space, before removing the stylet (‘late stylet removal’ (LSR)). If the needle has advanced too far, unintentional puncture of the anterior venous plexus can cause a blood-stained tap. ESR has therefore been adopted by some practitioners but is only backed by observational evidence [8, 9].

Other literature suggests no consistent evidence of significant benefit for: training in LP [11, 12]; seniority of practitioner [8, 9, 13–16]; sedation [13, 17]; local anaesthetic [8, 9, 12, 15, 18, 19]; and formulae for needle insertion depth (except certain subgroup analyses) [20]. Although there is growing evidence for ultrasound assistance [21–25], the equipment and training required has hindered widespread adoption. In comparison, sitting position and ESR are straightforward and already used by some practitioners.

### Aims

We aim to determine the optimal technique for lumbar puncture in newborn infants by evaluating the success rate, short-term clinical, resource, and safety outcomes of two modifications to traditional LP technique: infant position (sitting vs lying) and timing of stylet removal (early vs late).

This would be the first appropriately-powered RCT investigating neonatal LP technique and would therefore make a significant contribution to current knowledge.

## Methods/design

### Aim & Design

This is a pragmatic, multicentre, 2 × 2 factorial RCT to compare the proportion of infants with CSF obtained and RBC count < 10,000/mm^3^ on the first LP procedure when sitting position is adopted compared to lying position, and when ESR is performed compared to LSR. Other clinical decisions will be made according to local protocols. Clinicians undertaking LPs will receive training in the different trial techniques. Once a decision is made to perform an LP as part of routine care, parents of eligible infants will be approached to discuss consent. If given, randomisation will proceed. Staff will be advised to undertake 1–2 attempts (defined as the needle passing through the skin once) per ‘procedure’. The randomised technique will be used for up to two procedures. The requirement and timing for a second or any further procedures will be determined by the clinical team. Data will be collected from hospital records, and recorded on trial specific electronic case report forms (eCRFs). All samples sent to the laboratory will follow local site procedures, with relevant data from the laboratory reports inputted onto eCRFs. The trial rationale and design were closely discussed with and received input from local parent representatives from the local Support for the Sick Newborn And their Parents (SSNAP) charity [26].

The trial includes an internal pilot, which was based in centres recruiting the first 250 randomised infant participants in the first 8 months, in order to optimise study processes around recruitment, intervention delivery, training, and outcome assessments. The Trial Steering Committee (TSC) reviewed pilot data and have made recommendations and approved continuation.

**Fig. 1:**
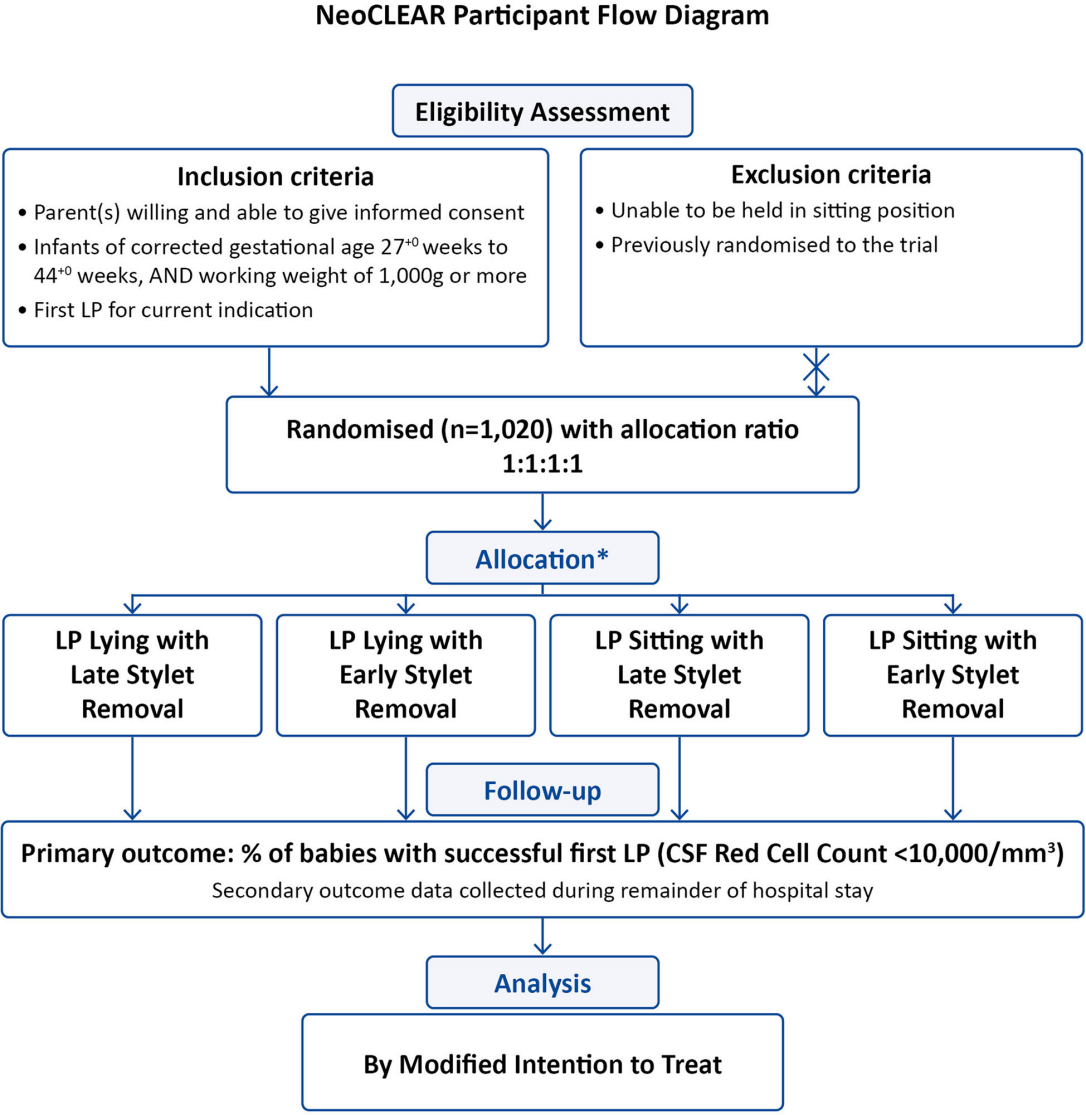
Study flowchart

The trial design is summarised in Fig. 1.

### Setting & participants

Infants who are having an LP in UK neonatal units and their maternity wards.

### Inclusion criteria

- Parent(s) willing and able to give informed consent
- Infants of corrected gestational age (CGA) from 27^+ 0^ weeks to 44^+ 0^ weeks, AND working weight of 1000 g or more
- First LP for current indication

### Exclusion criteria

- Unable to be held in sitting position (including infants intubated and mechanically-ventilated) or other clinical condition which is likely, in the opinion of the treating clinician, to make sitting difficult, or which is likely to be compromised by sitting (e.g. open gastroschisis).
- Previously randomised to the trial

### Schedule of study procedures

**Table:** 

Tasks	Visits			
	Screening	First LP	Further LPs (if needed)	Discharge
Eligibility confirmed	X			
Informed consent	X			
Randomisation	X			
STAI-S (Parent Questionnaire)^a^	X	X		
Data collection to eCRF	X	X	X	X
Serious/Adverse Event assessments		X	X	

^a^Internal pilot sites to complete following consent (before first LP) and within 48 h after first LP

### Primary outcome

The proportion of infants with successful first LPs (RBC count fewer than 10,000/mm^3^ in CSF on first procedure).

### Secondary outcomes

The following short-term clinical, resource and safety outcomes have been defined as:

- The proportion of infants with:
  - No CSF obtained, or pure blood/clotted, or blood-stained, or clear
  - CSF obtained and RBC count < 500, < 5000, < 10,000, or < 25,000 /mm^3^, or any RBC count
  - A CSF white blood cell (WBC) count not requiring a correction (whatever the RBC count)
- Total number of procedures and attempts performed per infant
- Proportion of infants diagnosed (by WBC count criteria, culture, Gram stain, and/or clinically) via CSF with:
  - Meningitis: WBC count 20 or more in CSF, or a true positive culture/Polymerase Chain Reaction (PCR) (if RBC count is ≥500, the WBC count will be reduced by 1 for every 500 RBC counts to give a ‘corrected’ WBC count)
  - Equivocal: WBC count (or corrected WBC) < 20, AND negative (or contaminated/incidental) culture and PCR with:
    - Either Polymorphonuclear leukocytes (PMN) > 2 (and RBC count < 500)
      OR
    - Organism found on Gram stain
  - Negative: WBC (or corrected WBC) count < 20, PMN ≤2 (if RBC count < 500), and negative (or contaminated/incidental) cultures, PCR, and Gram stain
  - Uninterpretable: No CSF obtained, or clotted, or CSF so bloody or insufficient that a cell count was impossible
- CSF WBC, RBC, corrected WBC counts, PMNs and lymphocytes from the clearest sample
- Time taken on first procedure from start of cleaning skin to removing needle at end of all attempts
- Infant movement on first procedure using a basic 4-point scale

### Outcomes related to cost and safety

- In all infants, according to CSF-defined and clinically-defined diagnostic criteria:
  - Duration of the antibiotic course
  - Length of stay in surviving infants
  - Immediate complications related to LP:
    - Cardiovascular instability, including oxygen saturations and heart rate
    - Respiratory deterioration (escalating respiratory support) post-LP
- For the pilot phase: parental anxiety (State Trait Anxiety Inventory - State Subscale (STAI-S) Questionnaire)

### Screening and recruitment

Infants who are having an LP will be screened for eligibility. Anonymised screening data will be recorded via the randomisation website for the Coordinating Centre to review rates of ineligibility and participant uptake rates.

Parents of eligible infants will be provided with both verbal and written information in a Parent Information Leaflet by their clinical team. Parents with legal parental responsibility will be approached to discuss the trial, answer any questions they may have and to request consent. Parents will have as much time as they need to consider and discuss the information with the research team, or other independent parties to decide whether to participate in NeoCLEAR. Written informed consent for the study will be obtained by a suitably qualified member of the study team. Parents completing the STAI-S will also be asked to provide written consent for their participation. Consent will be given at such time to allow randomisation and enable the clinical team to prepare for that procedure. Where this is not possible, the LP will not be delayed if the infant’s clinician deems any delay to be clinically unsound: in such cases, the infant would not be recruited to NeoCLEAR.

### Randomisation and blinding

Infants will be randomised 1:1:1:1 to one of the four arms: (1) Lying (lateral decubitus) position and LSR; (2) Lying position and ESR; (3) Sitting position and LSR, or (4) Sitting position and ESR, using a 24/7 secure web-based randomisation facility which will ensure balance between the groups. A telephone backup system will be available 24 h a day.

Stratified block randomisation will be used to ensure balance between the groups with respect to the collaborating hospital and CGA at trial entry (four groups: 27^+ 0^–31^+ 6^/32^+ 0^–36^+ 6^/37^+ 0^–40^+ 6^/41+ weeks). Where repeat (second) LPs are warranted, they will receive the same allocated technique. Multiple births will be randomised separately with their study ID numbers linked on the database prior to analysis.

A statistician independent of the trial will generate the randomisation schedule and the Senior Trials Programmer will write the web-based randomisation program; both will be independently validated. The implementation of the randomisation procedure will be monitored by the Senior Trials Programmer throughout the trial and reports will be provided to the Data Monitoring Committee (DMC).

This is an open-label trial as blinding of the practitioner and nursing staff to the allocated technique is not possible. The assessment of the primary outcome and major secondary outcomes will be based on laboratory tests (effectively blinded). Parents will not usually be told which technique their infant has been allocated, and are not routinely present for the procedure; however if they request this, it will be shared with them and they may observe the LP at the discretion of the practitioner.

### Baseline assessments

Trial entry data will include details to confirm eligibility and confirmation of parental written consent. During the pilot phase, after consent but before the first LP, the parent was asked to complete a 5-point Likert scale ‘How have you felt physically during the last couple of days?’, and STAI-S questionnaire [27, 28]. STAI-S is a well-validated measure consisting of 20 questions that identify how stressed/anxious someone is feeling at the time of assessment. All items are rated on a 4-point scale, the mean score will be used in analyses. Discontinuation of the STAI-S was recommended by the TSC based on a review of the pilot.

### Subsequent data collection

Most outcome data for this trial are routinely recorded clinical items obtained from the clinical notes. Non-routinely collected data includes procedural details such as time taken, infant movement, oxygen saturation and heart rate. No additional blood or tissue samples are required for this trial. All data will be collected using trial specific eCRFs. Outcome data will be collected until discharge home. Parents completing the STAI-S will be asked to complete a second questionnaire within 48 h following the first LP.

### Sample handling

As per best practice, all samples from any attempt should be sent to the microbiology laboratory for standard lab analyses, even if bloody or only 1–3 drops obtained, as culture may be informative.

### Description of procedure

Participating infants will require LP as part of routine care; NeoCLEAR randomisation will specify the technique used. Training will attempt to standardise other potential confounders, including needle type and analgesia. Practitioners will be trained and given written best practice guidance on technique, including recommendations for LP variables. However, adherence will not be mandatory, and will be captured in the trial eCRFs.

The first LP should be performed within the same shift as randomisation where possible, to minimise bias, in 1–2 attempts. If a second LP is required, the same technique should be employed. The need for any further procedures will be determined by the consultant.

### Safety reporting

An independent DMC will be established to review the study data and outcomes including safety reports of Serious Adverse Events (SAEs). The DMC will ensure the safety and wellbeing of the trial participants and make recommendations to the TSC regarding continuance of the study or modification of the protocol. The TSC will have ultimate responsibility for deciding whether the trial should be stopped on safety grounds. SAEs will be collected until the infant is discharged home, as SAEs occurring after this time point should not relate to the trial intervention. As parental participation is limited to the STAI-S questionnaire, no SAE recording will be conducted for this group.

The following are known, but rare, complications of LP: any occurrence following the LP should be reported as an SAE:

- Iatrogenic meningitis
- Iatrogenic haemorrhage: spinal haematoma (symptomatic), intraventricular, intracerebral and subarachnoid haemorrhage
- Cerebral herniation
- Nerve damage

The full protocol and Guidance Sheets provided to sites also pre-specify expected SAEs that are foreseeable in the trial population, and will not be reported unless thought to be causally related to trial procedures.

All unforeseeable SAEs occurring after consent until discharge home must be reported.

### Discontinuation and withdrawal

Parents have the right to withdraw consent for any aspect of the study, including their infant’s future procedures and data collection, or their own questionnaire completion. The treating clinician may discontinue a participant if they consider it to be in the best interests of the infant’s health and wellbeing.

### Definition of end of study

The end of the trial will be defined as the date when the trial database is locked. An end of trial declaration will be made to REC.

### Project management

The study is sponsored by the University of Oxford. The trial will be run by the NPEU CTU, based at the University of Oxford and the CI. On a day-to-day basis, the trial will be run by a Project Management Group (PMG) according to NPEU CTU SOPs and will be subject to audit and inspection. The core PMG will meet every month, either remotely or face-to-face. An extended PMG (Co-Investigator Group) will meet regularly to troubleshoot, review progress and forward plan. The PMG reports to the TSC.

The trial will be overseen by the TSC which will have ultimate responsibility for considering and, as appropriate, acting on the recommendations of the DMC. The TSC will include an independent chair, at least one clinician, statistician and Patient and Public Involvement (PPI) representative, and the CI. The TSC will meet at least annually and review the progress of the trial.

The DMC will be independent of the study and the TSC. The DMC will review the progress of the trial and interim analysis at least annually, and make recommendations on the conduct of the trial to the TSC.

### Patient and public involvement

Patient and public representatives have been extensively involved in trial planning, grant/protocol writing, and preparing study materials. Advice from two PPI co-applicants from local charity SSNAP [26] included aspects of protocol design, wording in parent-facing documentation, as well as ongoing recruitment initiatives. Contributions on documentation were also received from Bliss baby charity [29]. A lay person is a member of the TSC, and will be involved in trial oversight and dissemination of findings.

### Statistics and analysis

#### Sample size and power calculation

Four hundred eighty-three infants are required for each arm of the main comparison (sitting vs lying position and ESR vs LSR), to detect a 10% absolute difference (from 59 to 69%) in the proportion of infants with successful LPs, with 90% power, a 5% two-sided significance level and assuming (based on expert opinion and the lack of external evidence) no interaction effect between infant position and timing of stylet removal. Allowing for 5% attrition, the required recruitment target is 1020 (255 in each of the four groups). The 59% control event rate was derived from local prospective cohort data [30] and corroborated by published UK data [20]. We estimate 50% parental uptake rate and minimal ‘loss to follow-up’ as all data will be collected pre-discharge.

Modelling suggests we will need 10 local Neonatal Units and Neonatal Intensive Care Units to recruit enough infants within 24 months, with a recruitment rate of around 5 infants per centre per month assuming: staggered starts (two centres per month), and each centre taking 4 months to reach stable recruitment rate. PMG will implement recruitment initiatives and additional centres, based on actual recruitment.

Our inclusion/exclusion criteria are designed to minimise protocol deviations by only including infants who can be sat up. Only sparse safety data is available for LPs < 27 weeks and < 1000 g; hence these infants will be excluded from our trial.

#### Description of statistical methods

Outcomes for participants will be analysed in the groups to which they are assigned regardless of deviation from the protocol or allocation received, but will be excluded from the analysis if no LP procedure was received (modified intention-to-treat analysis). To assess the effect of position we will compare groups (1–Lying/LSR) plus (2–Lying/ESR) with groups (3–Sitting/LSR) plus (4–Sitting/ESR), and to assess the effect of the timing of stylet removal we will compare groups (1) plus (3) with groups (2) plus (4). We will calculate the risk ratio (95% Confidence Interval (CI)) for the primary outcome (and all other dichotomous outcomes), the mean difference (95% CI) for normally distributed continuous outcomes or the median difference (95% CI) for skewed continuous outcomes. Absolute risk difference and CIs will also be calculated for tested dichotomous clinical outcomes (to be presented in a supplementary appendix). Groups will be compared using regression analysis, adjusting for the stratification variables used at randomisation. Both crude and adjusted estimates will be presented but the primary inference will be based on the adjusted analyses. Adjusted risk ratios will be estimated using log-binomial regression, or a Poisson regression model with a robust variance estimator in the event of non-convergence. Linear regression will be used for normally distributed outcomes and quantile regression for skewed continuous outcomes.

With a view to minimising the effects of multiple testing given the number of procedures and attempts performed for each infant, and correlation between some outcomes, statistical inference will be restricted to a predefined list of tested outcomes. Summary data by trial arm will be provided for all other outcomes but statistical tests (or the calculation of CIs) will not be performed. A complete list of outcomes that will be tested and not tested statistically are provided in a supplementary appendix.

Full details of the statistical analysis will be documented in the Statistical Analysis Plan.

#### Interaction testing

The interaction between sitting/lying position and the timing of stylet removal will be investigated for the primary outcome, though we acknowledge that the trial is not powered to detect an interaction effect. A descriptive multi-arm analysis will also be presented for the primary outcome, other tested outcomes, and baseline characteristics (i.e. for each of the four trial arms) as supplementary information [31].

#### Interim data monitoring

Interim data monitoring will be carried out by the DMC at least annually. The DMC Charter will include details about timings of reviews, contents of the reports and any stopping guidelines.

## Discussion

### Participant risks and benefits

All trial participants will be having an LP as part of routine clinical care. All techniques are in current use and none is proven to be more or less safe than another. No infants are expected to be placed at risk as a result of being in the trial. Serious complications of LP are rare. Some complications can be prevented by avoiding LPs in infants with contraindications such as coagulopathy; or mitigated, e.g. with sterile technique. Potential benefits of trial participation include: an optimised analgesia protocol; all infants will have heart rate and oxygen saturations monitored during the procedure.

### Parent questionnaire

Parents completing the STAI-S questionnaire will be asked about their feelings and levels of anxiety which are sensitive and upsetting topics. Our PPI group and previous research has indicated that in stressful situations, parents benefit from being given the opportunity to express their feelings. Completion of the questionnaire will not be pursued if a parent becomes distressed when completing it.

### Consent

Ideally, consent will be given at such time to allow randomisation and enable the clinical team to prepare for that procedure. However, in instances where this is not possible, the LP will not be delayed if, in the opinion of the infant’s clinician, any delay would be deemed clinically unsound: in such cases, the infant would not be recruited to the trial.

### Participant confidentiality

The infants’ and parents’ names will be shared with NPEU CTU via the consent form. Parents of infants participating in the trial will be informed of, and provide consent to this. No other personal identifiable information will be shared outside of the site.

Overall responsibility for ensuring that each participant’s information is kept confidential lies with the Sponsor. All paper documents will be stored securely and kept in strict confidence in compliance with current data regulations. Data collected on the (e)CRFs will be stored in an electronic database held by the Trial Coordinating Centre in which the participant will be identified only by a trial specific number.

Following trial completion and report publication, data will be archived in a secure physical and electronic location with restricted access.

### Participant remuneration

No financial or material incentive or compensation will be provided to parents for enrolling their infants in this trial.

### Dissemination

The success of the trial depends on a large number of research nurses, neonatal nurses, doctors, and parents. Credit for the trial findings will be given to all who have collaborated and participated in the trial including all local coordinators and collaborators, members of the trial committees, the NeoCLEAR Coordinating Centre and trial staff. Authorship at the head of the primary results paper will take the form “[name], [name] and [name] on behalf of the ‘The NeoCLEAR Collaborative Group’”. All contributors to the trial will be listed at the end of the main paper, with their contribution identified. It is the intention of the NeoCLEAR Collaborative Group to present data at national/international conferences and to publish open-access, peer-reviewed articles, including the analysis of key outcomes. The NPEU Clinical Trials Unit and local newborn charity will disseminate the results at conferences and coordinate press releases, website promotion, social and other media interest.

## Supplementary information

**Additional file 1.** Supplementary appendix: List of tested and untested outcomes.

## Data Availability

Not applicable.

## Abbreviations

CGA: Corrected gestational age
CSF: Cerebrospinal fluid
CTU: Clinical Trials Unit
DMC: Data Monitoring Committee
eCRFs: Electronic Case Report Forms
ESR: Early stylet removal
HRA: Health Research Authority
LP: Lumbar puncture
LSR: Late stylet removal
NIHR: National Institute for Health Research
NPEU: National Perinatal Epidemiology Unit
PCR: Polymerase Chain Reaction
PMNs: Polymorphonuclear Leukocytes
PPI: Patient and public involvement
RBC: Red Blood Cell(s)
RCT: Randomised controlled trial
REC: Research Ethics Committee
SAE: Serious Adverse Event
SSNAP: Support for the sick newborn and their parents
STAI-S: State-Trait Anxiety Inventory Subscale
TSC: Trial Steering Committee
WBC: White Blood Cell(s)

## References

[1] Heath PT, Balfour G, Weisner AM, Efstratiou A (2004). Group B streptococcal disease in UK and Irish infants younger than 90 days. Lancet..

[2] Galiza EP, Heath PT (2009). Improving the outcome of neonatal meningitis. Curr Opin Infect Dis.

[3] Cotten CM, Taylor S, Stoll B, Goldberg RN, Hansen NI, Sánchez PJ (2009). Prolonged duration of initial empirical antibiotic treatment is associated with increased rates of necrotizing Enterocolitis and death for extremely low birth weight infants. Pediatrics..

[4] Patel SJ, Saiman L, Stanley M (2010). Antibiotic resistance in NICU pathogens: mechanisms, clinical impact, and prevention including antibiotic stewardship. Clin Perinatol.

[5] Mukherjee A, Davidson L, Anguvaa L, Duffy DA, Kennea N (2015). NICE neonatal early onset sepsis guidance: greater consistency, but more investigations, and greater length of stay. Arch Dis Child Fetal Neonatal Ed.

[6] Baziomo J, Kremp O, Leke L, Mahomedaly H, O’Cheik A, Eb F (1995). Analyse rétrospective de 1331 échantillons de liquide céphalorachidien chez le nouveau-né suspect d’infection. Arch Pediatr.

[7] Greenberg RG, Smith PB, Cotten CM, Moody MA, Clark RH, Benjamin DK (2008). Traumatic lumbar punctures in neonates. Pediatr Infect Dis J.

[8] Baxter AL (2006). Local anesthetic and Stylet styles: factors associated with resident lumbar puncture success. Pediatrics..

[9] Nigrovic LE, Kuppermann N, Neuman MI (2007). Risk factors for traumatic or unsuccessful lumbar punctures in children. Ann Emerg Med.

[10] Hart C, Thompson A, Moriarty P (2016). Is the lateral decubitus position best for successful paediatric lumbar puncture?. Arch Dis Child..

[11] Srivastava G, Roddy M, Langsam D, Agrawal D (2012). An educational video improves technique in performance of pediatric lumbar punctures. Pediatr Emerg Care.

[12] Kessler D, Auerbach M, Pusic M, Tunic M, Foltin J (2011). A randomized trial of simulation-based deliberate practice for infant. Simul Healthc.

[13] Carraccio C, Feinberg P, Hart LS, Quinn M, King J, Lichenstein R (1996). Lidocaine for lumbar punctures - a help not a hindrance. Arch Pediatr Adolesc Med.

[14] Glatstein MM, Zucker-Toledano M, Arik A, Scolnik D, Oren A, Reif S (2011). Incidence of traumatic lumbar puncture: experience of a large, tertiary care pediatric hospital. Clin Pediatr (Phila).

[15] Howard SC, Gajjar AJ, Cheng C, Kritchevsky SB, Somes GW, Harrison PL (2002). Risk factors for traumatic and bloody lumbar puncture in children with acute lymphoblastic leukemia. JAMA..

[16] Pinheiro JM, Furdon S, Ochoa LF (1993). Role of local anesthesia during lumbar puncture in neonates. Pediatrics..

[17] Derakhshanfar H, Kordi MM, Amini A, Shojahee M (2013). A comparative study on the sedative effect of oral midazolam and oral chloral hydrate medication in lumbar puncture. Acta Med Croat.

[18] Porter FL, Miller P, Sessions F, Marshall RE (1991). A controlled clinical trial of local anesthesia for lumbar punctures in newborns. Pediatrics..

[19] Shenkman A, Fukuda J, Benincasa G, Ruiz M, McSherry K, Ahmad K (2002). Incidence of traumatic lumbar puncture in children treated with EMLA® at a pediatric emergency room. Pediatr Emerg Care.

[20] Murray MJ, Arthurs OJ, Hills MH, Kelsall W (2009). A randomized study to validate a midspinal canal depth nomogram in neonates. Am J Perinatol.

[21] Kim S, Adler DK (2014). Ultrasound-assisted lumbar puncture in pediatric emergency medicine. J Emerg Med.

[22] Lam SHF, Lambert MJ (2015). In reply: ultrasound-assisted lumbar puncture in pediatric patients. J Emerg Med..

[23] Özdamar E, Özkaya A, Güler E, Cantay B, Karabel N, Göksügür Y (2017). Ultrasound-assisted lumbar puncture in pediatric emergency department. Pediatr Emerg Care.

[24] Neal JT, Kaplan SL, Woodford AL (2017). The effect of bedside ultrasonographic skin marking on infant lumbar puncture success: a randomized controlled trial. Ann Emerg Med.

[25] Gorn M, Kunkov S, Crain EF (2017). Prospective investigation of a novel ultrasound-assisted lumbar puncture technique on infants in the pediatric emergency department. Acad Emerg Med.

[26] Support for the sick newborn and their parents (SSNAP): https://www.ssnap.org.uk.

[27] Spielberger CD (1989). State–trait anxiety inventory: a comprehensive bibliography.

[28] Elliott TR, Shewchuk RM, Richards JS (2001). Family caregiver problem solving abilities and adjustment during the initial year of the caregiving role. J Couns Psychol.

[29] Bliss – for babies born premature or sick: www.bliss.org.uk.

[30] Marshall AS, Sadarangani M, Roehr CC, Anthony M. Champagne, Rosé, or a Bloody Mary? – a prospective audit of lumbar punctures in a tertiary neonatal unit. European Society for Paediatric Infectious Diseases Annual Meeting 2015; ESPID-0989, Oral Presentation.

[31] Kahan BC, Tsui M, Jairath V, Scott AM (2020). Reporting of randomized factorial trials was frequently inadequate. J Clin Epidemiol.

[32] NeoCLEAR study sites: https://www.npeu.ox.ac.uk/neoclear/sites.

