# Supplementary material for: Study protocol: NeoCLEAR: Neonatal Champagne Lumbar punctures Every time – An RCT: a multicentre, randomised controlled 2 × 2 factorial trial to investigate techniques to increase lumbar puncture success
Source: BMC Pediatr. 2020 Apr 15;20:165. doi: 10.1186/s12887-020-02050-8 (PMC7160994; doi:10.1186/s12887-020-02050-8)
Supplement: Supplementary file 1 — Additional file 1. Supplementary appendix: List of tested and untested outcomes. [file 12887_2020_2050_MOESM1_ESM.docx]

Supplementary appendix: List of tested and untested outcomes

Due to the multiple number of procedures and attempts performed for each infant, and correlation between some outcomes, statistical inference will be restricted to a predefined list of tested outcomes. Summary data by trial arm will be provided for all other outcomes but statistical tests (or the calculation of confidence intervals) will not be performed.

Primary outcome – tested

Proportion of infants with CSF obtained and RBC count <10,000/mm^3^ on the first LP procedure (any attempt).

Secondary clinical outcomes – tested

*Note: clearest is defined as follows: getting a sample is better than none; in a sample, clear CSF is best > blood-stained > pure blood/clotted; CSF sent to the lab is better than not sent; If the lab are able to do a microscopy (not clotting, and reporting RBC and WBC counts) it is better than not; if the lab are able to do a microscopy then the lower the RBC count, the better.*

- Proportion of infants with:
  - No CSF obtained, or pure blood/clotted, or blood-stained, or clear CSF from clearest sample of the first procedure – any attempt
  - CSF obtained with any RBC count on first procedure – any attempt
  - CSF obtained with WBC count not requiring correction on first procedure – from any attempt (WBC count <20 whatever the RBC count, or RBC count <500)
- Proportion of infants diagnosed by the clinical team at discharge – in relation to their LP(s) – with:
  - Definite/probable meningitis
  - Possible meningitis or equivocal CSF result
  - Negative CSF result
  - Uninterpretable CSF result (e.g. very high RCC or clotted CSF)
  - No CSF obtained
- WBC count, RBC count, corrected WBC count, PMN, and lymphocytes from clearest CSF sample
- Total number of procedures performed per infant
- Total number of attempts performed per infant
- Time taken to complete the first procedure, from start of cleaning skin to removing needle at end of all attempts
- Level of infant struggling movement on first attempt of first procedure

Secondary clinical outcomes – untested

- For: the first attempt of the first procedure; any attempt of first procedure (if not in ‘tested’ outcomes); the first or second procedure:
  - CSF appearance (Clear CSF/blood-stained/pure blood or clotted/no sample obtained)
  - CSF obtained and any RBC count
  - CSF obtained and RBC count <500/mm^3^
  - CSF obtained and RBC count <5,000/mm^3^
  - CSF obtained and RBC count <10,000/mm^3^
  - CSF obtained and RBC count <25,000/mm^3^
  - CSF obtained with WBC count not requiring correction (WBC count <20 whatever the RBC count, or RBC count <500)
- Number of attempts for first and second procedure per infant
- Proportion of infants diagnosed by CSF from first two procedures with:
  - Meningitis: WBC count 20 or more in CSF, or a true positive culture/PCR (if RBC count is ≥500, the WBC count will be reduced by 1 for every 500 RBC counts to give a ‘corrected’ WBC count)
  - Equivocal: WBC count (or corrected WBC) <20, AND negative (or contaminated/ incidental) culture and PCR with:
    - either PMN >2 (and RBC count <500)
    - OR organism found on Gram stain
  - Negative: WBC (or corrected WBC) count <20, PMN ≤2 (if RBC count <500), and negative (or contaminated/ incidental) cultures, PCR, and Gram stain
  - Uninterpretable: No CSF obtained, or clotted, or CSF so bloody or insufficient that a cell count was impossible

Cost outcomes – tested

- Duration of the antibiotic course from trial entry to discharge home
- Length of stay in hospital in surviving infants from trial entry until discharge home

Safety outcomes – tested

- Immediate complications related to first procedure:
  - Procedure abandoned due to cardiovascular deterioration
  - Infant’s lowest oxygen saturation (%)
  - Infant’s lowest heart rate (BPM)
  - Infant’s highest heart rate (BPM)
  - Respiratory deterioration post-LP (requirement for escalating respiratory support within 1 hour of the LP)

Safety outcomes – untested

- Immediate complications related to second procedure:
  - Procedure abandoned due to cardiovascular deterioration
  - Respiratory deterioration post-LP (requirement for escalating respiratory support within 1 hour of the LP)
